# Subclinical toxicity of calcineurin inhibitors in repeated protocol biopsies: an independent risk factor for chronic kidney allograft damage

**DOI:** 10.1111/j.1432-2277.2009.00995.x

**Published:** 2010-04

**Authors:** Karel Krejčí, Tomáš Tichý, Miroslav Hrubý, Pavel Horák, Hana Ciferská, Vladko Horčička, Pavel Štrebl, Sadek Al-Jabry, Petr Bachleda, Josef Zadražil

**Affiliations:** 1 3rd Department of Internal Medicine and Nephrology, University Hospital OlomoucOlomouc, Czech Republic; 2 Institute of Pathology, University Hospital OlomoucOlomouc, Czech Republic; 3 2nd Surgical Department and Transplant Centrum, University Hospital OlomoucOlomouc, Czech Republic

**Keywords:** Banff chronicity score, calcineurin inhibitors, kidney transplantation, protocol biopsy, renal allograft damage, subclinical nephrotoxicity

## Abstract

The purpose of the prospective study was to determine the prevalence of subclinical toxicity of calcineurin inhibitors (CI) in repeated protocol renal allograft biopsies and to assess its impact on the development of chronic graft changes. A total of 424 biopsies were conducted in a cohort of 158 patients; of these biopsies, 158 were in the third week, 142 were in the third month and 124 were in the first year after transplantation. Histological signs of toxicity occurred in the third week in 33 (20.1%) patients, with persistence after CI dose reduction in the third month in 27 (19.0%) and in the first year in 23 (18.5%) patients. Of the toxic changes, 52% were clinically silent. At the end of the one-year follow-up, both subclinical and clinically manifest toxicity resulted in a similar progression of chronic changes quantified by Banff chronicity score and they significantly differed from the control group (*P*< 0.05). Subclinical toxicity affects a significant percentage of grafts; it occurs independently of dosage, blood level and type of applied CI. It is associated with the progression of chronic changes as early as in the first year after transplantation and represents an independent risk factor for chronic allograft damage. We report here our clinical approach to toxicity.

## Introduction

In light of large multicenter studies, the time limited renal graft function remains one of the key problems of current transplantology [1]. Despite the dropping prevalence of clinically manifest acute rejection and despite the active approach to diagnostics and treatment of subclinical rejection, adequate improvement in the long-term graft survival has not been achieved [2].

The chronic nephrotoxic effects of calcineurin inhibitors (CI) associated with the development of chronic parenchymal changes play a major role in the pathogenesis of late dysfunction [3]. The effort to achieve maximum CI dose reduction and careful monitoring of its serum levels are very important in the prevention of the development of these irreversible changes. The administration of CI, however, is complicated by the much varied pharmacokinetics, narrow therapeutic range, and individual sensitivity to toxic effects [4]. For these reasons, CI serum levels often do not correlate with the scope of kidney damage and the manifestation of toxicity may be nonspecific, or the toxic changes may be, especially in the first months after transplantation, clinically silent. The impact of this subclinical toxicity on the progression of chronic graft changes is likely, yet available data are limited [5].

The purposes of our prospective study were to evaluate the prevalence of subclinical toxicity in repeated protocol biopsies conducted in the third week, third month and the first year after the transplantation and to assess its impact upon the development of chronic graft changes at the end of the one-year monitoring. Concurrently, we strove to determine whether there was any difference between the two applied CI – cyclosporin A and tacrolimus – in terms of their ability to affect the development of toxic and, subsequently, chronic changes.

## Patients and methods

### Study design

This was a prospective, nonrandomized, single-arm, one-centre study which was conducted according to ICH/GCP guidelines and supported by a grant and research project. The trial was approved by the Local Ethics Committee and all patients gave their written and dated informed consent before inclusion in the study. In total, 424 protocol graft biopsies were conducted in a cohort of 158 patients with new transplants. The population consisted of 88 women and 70 men aged 18–73 years, with mean age of 51.2 ± 13.1 years. All patients received a kidney from a deceased donor; for six patients it was their second transplantation and in one case, it was a third transplantation. Individuals included in the study were treated by a triple combination immunosuppressive therapy including cyclosporin A or tacrolimus, the antimetabolite azathioprine or the mycophenolate mofetil and prednison. In the induction therapy, we used mostly methylprednisolon (Day 0–500 mg i.v. perioperatively, Day 1–250 mg i.v.), as well as anti-thymocyte globulin, and, where appropriate, anti-IL2-R monoclonal antibody in recommended dosage. The initial CI dose was administered 4 h before the operative procedure, in patients indicated for treatment with cyclosporin A it was 3 mg/kg, and 0.1 mg/kg for tacrolimus. Further CI dosage was maintained with regard to the levels and time from transplantation (Table 1). Where histological signs of toxicity were seen, the dose was reduced by 25–50% with a view to the initial CI level. Patients receiving azathioprine or mycophenolate mofetil were kept at the dosage of 1.5 mg/kg/day and 2 g/day, respectively, with dose reduction upon the incidence of adverse events or complications associated with this therapy. All patients were given prednison in the dose of 30 mg from Day 2 to day 14, 20 mg from Day 15 to Month 2 after the transplantation, and then 15 mg from Month 3 to month 5 and 10 mg from Month 6 to month 12.

**Table 1:** Target levels of calcineurin inhibitors in dependence on time offset after transplantation.

Level CI (μg/l)		Time offset after transplantation
CsA-C 0^a^	250–350	Days 1–6
	200–300	Days 7–30
	150–200	Months 2–6
	100–200	>6months
CsA-C 2^b^	1300–1800	Month 1
	1200–1600	Month 2
	1000–1400	Month 3
	900–1200	Months 4–6
	700–1000	Months 7–12
FK-C 0^c^	10–20	Days 1–14
	10–15	Days 15–30
	5–10	>30 days

CI, calcineurin inhibitor.

a CsA-C 0, trough level of cyclosporin A.

b CsA-C 2, 2-hour cyclosporin A post-dose level.

c FK-C 0, trough level of tacrolimus.

### Protocol biopsies and histological evaluation

Biopsies were carried out in the third week, third month and the first year after the transplantation. A total of 158 biopsies were performed in the third week, 142 in the third month and 124 in the first year. The procedure was conducted using the Magnum MG 1522 automatic biopsy system and Magnum 14 G/13 cm biopsy needles. Two samples were taken under ultrasound control from the lower pole of the graft. The histological evaluation of the biopsy specimen used the modified Banff 97 classification [6,7], performed by a single pathologist familiar with the clinical progress. A sample containing at least seven glomeruli and at least one artery was rated as adequate. Following sampling, the tissue was preserved in 10% formalin, processed in a normal manner and embedded in paraffin. Histological samples were processed to slices of 3–4 micron thickness and then stained with haematoxylin eosin on three slides, using the Periodic Acid-Schiff method on three slides and using Masson trichrome on one slide. Immunohistochemical analysis using polyclonal antibody was employed for the detection of C4d positivity in peritubular and glomerular capillaries. A minimum of 12 slices from each sample were evaluated.

Isometric vacuolization of the tubular epithelium, necrotic arteriole myocytes with early deposition of eosinophilic globular material in arteriole walls, the presence of fibrinoid arteriole necrosis, and, eventually, thrombotic microangiopathy with concurrent exclusion of acute rejection as well as accelerated hypertension were considered manifestations of acute CI toxicity. Arteriole myocytes vacuolization, a whirling myocytes pattern, tubular microcalcifications and arterial mucoid intimal thickening were not classified as certain signs of toxicity on their own, but merely in combination. The signs of chronic toxicity were considered the striped cortical fibrosis, tubular atrophy, nodular and circular arteriolohyalinosis and hyperplasia of the juxtaglomerular apparatus. Focal global and segmental glomerulosclerosis without basal membrane reduplication and without podocyte hyperplasia was classified as the sign supporting the diagnosis, as well as tubular microcalcification. None of the above mentioned signs occurred as isolated in the samples.

### Laboratory analysis

Immediately before each biopsy, laboratory parameters of the transplanted kidney function were established – glomerular filtration rate by Nankivell and serum creatinine concentration by enzymatic method. A serum creatinine level of 125 μmol/l was determined as the upper limit of normal values (ULN). The function of the graft was considered stabilized in serum creatinine fluctuations <10% at least within two weeks prior to the protocol biopsy.

Cyclosporin A and tacrolimus levels were established as fasting levels 12 h following prior application. In half of patients taking cyclosporin A, they were established also two hours following oral application of cyclosporin A (C 2 level). The analytical method employed was fluorescence polarization immunoassay (FPIA ABBOTT Laboratories).

### Inclusion and exclusion criteria and BCS monitoring

Based on the results of graft function established through the biopsies conducted in the third week after transplantation, four groups were detached from the study population to serve the object of our interest. Patients with normal histological findings and stabilized graft function were included in the comparison group (NORMAL). Subjects with histological signs of CI toxicity, but normal and stabilized graft function were placed in the subclinical toxicity group (S-TOX). Those with histological evidence of toxicity and an impaired graft function without any other cause of dysfunction were included in the clinically manifest toxicity group (M-TOX). Patients with histological signs of toxicity, independent of the graft function, were also evaluated (S+M-TOX group). The CI level was not a criterion affecting the inclusion of the patient in any of the monitored groups. Two CI patient subgroups were defined in each group – one with cyclosporin A (CsA subgroup), and one with tacrolimus (FK subgroup).

The patients who had medical and technical contraindications and limitations for biopsy and who refused to sign the informed consent form were not included in the study or were subsequently withdrawn from the study. The same procedure was applied to the identification of an acute rejection in any form (antibody or T-cell mediated, incl. borderline rejection changes and subclinical acute rejection), recurrent and de novo glomerulonephritis, acute pyelonephritis and CMV infection at the beginning and thereafter at any time during the study, for the reason of potential influence upon chronic damage progression. All of the studied groups and subgroups were monitored for the development of chronic changes (ci, ct, cg, cv) using Banff chronicity score (BCS) in the course of the one-year monitoring, and cross-comparisons of a number of other donor, donor kidney and recipient parameters were carried out. Chronic changes identified in the third week were, in otherwise uncomplicated post-transplantation course, considered as a transfer from donor. The S-TOX and M-TOX groups were separately evaluated for potential persistence of toxic signs in subsequent biopsy in relation to the previous CI dose reduction.

### Statistical analysis

For the statistical analysis of the results the spss for Windows, version 15.0 software (SPSS Inc., Chicago, IL, USA) was used. Quantitative elements in this work were described using the statistics average ± SD, category elements using frequency distribution. Chi-square test or Fisher’s exact test (suitable for low frequencies) was used for the comparison of individual groups in categorical elements. In terms of quantitative elements, the groups were compared using analysis of variance and LSD *post hoc* tests. Group comparisons employed, depending on the nature of data, either nonparametric Mann–Whitney U-test (in the case of abnormal distribution of values or presence of extreme values in the data) or two-tailed *t*-test. Test results were labelled as significant where the level of statistical significance *P*< 0.05 was achieved.

## Results

### Prevalence and persistence of subclinical and clinically manifest toxicity

Table 2 documents four groups isolated from the study population for the next evaluation in the third week and the degree of persistence of these baseline parameters in the course of the one-year monitoring. In the third week, a surprisingly low percentage of normal histological findings were seen – only in 30 patients (19.0%). The identification of CI toxicity, on the contrary, was rather high – clinically manifest in 16 patients (10.1%) and subclinical in 17 patients (10.8%), as well as the high persistence of toxic changes in the course of the one-year monitoring in both toxicity groups, regardless of the repeated reductions in the CI dose. In a maximum of 12% of patients with histological evidence of toxicity, the elevation of serum creatinine was seen at the same time (Table 3). Patients who no longer complied with the required criteria and in whom no further biopsy had been performed were excluded from the studied groups in the third month and in the first year. With a view to this, in the NORMAL group, 21 patients continued to participate in the study in the third month; 12 in the CyA subgroup and nine in the FK subgroup, and at the end of the one-year monitoring, 14 patients were evaluated in this group; eight in the CyA subgroup and six in the FK subgroup. In the S-TOX and M-TOX groups, 14 and 13 patients respectively continued to participate in the study in the third month (nine in the CyA subgroup and five in the FK subgroup and seven in the CyA subgroup and six in the FK subgroup), and at the end of the one-year monitoring, the number of evaluated patients was 12 and 11 respectively (seven in the CyA subgroup and five in the FK subgroup; and six in the CyA subgroup and five in the FK subgroup). Of the total number of seven patients with re-transplantations who had a biopsy in the third week, only three patients continued to participate in the study in the third month. These were evenly distributed across studied groups and the impact of re-transplantation upon the development of monitored parameters could thus be disregarded.

**Table 3:** Graft function in relation to level of calcineurin inhibitor at patients with subclinical and manifest toxicity.

	3rd week *n* (%)	3rd month *n* (%)	1st year *n* (%)
S+M-TOX group	33 (20.9)	27 (19.0)	23 (18.5)
Elevated creatinine	16 (48.5)	13 (48.1)	11 (47.8)
Elevated CI	11 (33.3)	7 (25.9)	3 (13.0)
CI and creatinine elevated	4 (12.1)	3 (11.1)	1 (4.3)
Creatinine in a normal range	17 (51.5)	14 (51.9)	12 (52.2)
CI in a normal range	22 (66.7)	20 (74.1)	20 (87.0)
CI and creatinine in a normal range	10 (30.3)	10 (37.0)	10 (43.5)

Data are number (%).

S+M-TOX group, composite group with subclinical and manifest toxicity; CI, trough level of calcineurin inhibitor.

**Table 2:** Four detached groups from the study population and rate of persistency of these baseline histological findings during one year follow-up.

	3rd week		3rd month		1st year	
Group	*n* (%)	Creatinine (μmol/l) mean ± SD	*n* (%)	Creatinine (μmol/l) mean ± SD	*n* (%)	Creatinine (μmol/l) mean ± SD
NORMAL	30 (19.0)	102.5 ± 18.9	21 (14.8)	111.8 ± 21.3	14 (11.3)	105.0 ± 15.7
S-TOX	17 (10.8)	98.6 ± 15.1	14 (9.9)	101.4 ± 14.4	12 (9.7)	103.0 ± 13.6
M-TOX	16 (10.1)	207.8 ± 107.2	13 (9.2)	161.8 ± 29.4	11 (8.9)	147.7 ± 14.7
S+M-TOX	33 (20.9)	151.6 ± 92.6	27 (19.0)	130.5 ± 38.1	23 (18.5)	124.4 ± 26.7

Data are mean ± standard deviation (SD) or number (%).

NORMAL, normal histological finding; S-TOX, subclinical toxicity of calcineurin inhibitors; M-TOX, manifest toxicity of calcineurin inhibitors; S+M-TOX, composite group with subclinical and manifest toxicity.

### BCS and serum creatinine evaluation

**Figure 1:**
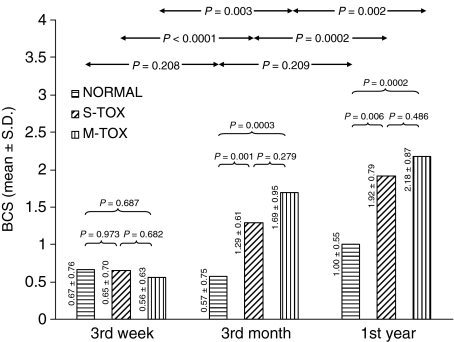
Banff chronicity score (BCS) of monitored groups and its evolution during one year follow-up (NORMAL, normal histological finding; S-TOX, subclinical toxicity of calcineurin inhibitors; M-TOX, manifest toxicity of calcineurin inhibitors). Data are mean ± standard deviation (SD) and level of statistical significance *P*.

The assessment of the impact of persisting subclinical toxicity on the progression of chronic graft changes employed a comparison of the BCS development in this group with findings in the NORMAL and M-TOX groups. Earlier, groups had been compared for a number of baseline donor and recipient parameters, kidney graft parameters and other factors potentially influencing the development of chronic changes (Table 4). Similar comparison was then conducted again in the third month and the first year after transplantation (data not presented), always along with the protocol biopsy. No significant difference among groups was found in the monitored parameters and no other immunopathogenic conditions resulting in systemic impairment have occurred in the patients in the course of the study. Hence, the progression of chronic changes could be assessed in relation to the presence or absence of toxicity. Figure 1 documents the development of the BCS of evaluated groups. In the NORMAL group, the increase was only moderate and did not reach statistical significance at the end of the one-year monitoring. In the M-TOX group, a significant increase in the BCS was identified as early as from the third week to the third month after transplantation, and a further significant increase occurred from the third month to the first year after transplantation. Surprisingly, the similar progression of chronic changes was also seen in patients with subclinical toxicity. Moreover, BCS values in this group did not differ significantly from the values found in the M-TOX group.

**Table 4:** Baseline characteristics and others monitored parameters of the initial cohort (*n*= 158).

Characteristics of the donor	
Age (years) (mean ± SD)	47.6 ± 19.3
Gender (Male/Female)	57.6/42.4
Race (W/B/A/H)	100/0/0/0
Deceased/living	100/0
Cause of death (T/K/I/H)	41.2/54.4/1.9/2.5
Number of mismatches – HLA-A (0/1/2)	8.2/54.4/37.4
– HLA-B (0/1/2)	5.3/45.6/49.1
– HLA-DR (0/1/2)	15.8/58.2/26.0
HLA-A, B, DR mismatch (mean ± SD)	3.7 ± 1.0
Blood group (A/O/B/AB)	39.2/42.4/14.6/3.8
Vasopressor administration (Y/N)	34/66
Diuresis (litres) (mean ± SD)	2.59 ± 2.34
Preservation solution (C/EC/UW/CU)	0/5.7/17.1/77.2
Serum creatinine (mean ± SD)	97.62 ± 41.54
CMV IgG (P/N)^a^	64.6/35.4
CMV IgM (P/N)	7.6/92.4
Characteristics of the recipient	
Age (years) (mean ± SD)	51.2 ± 13.1
Gender (Male/Female)	44.3/55.7
Race (W/B/A/H)	100/0/0/0
Re-transplantation (Y/N)	4.4/95.6
Recipient diagnosis (GN/TIN/DM/PCKD/AS/T)	40.5/30.4/7.6/21.5/0
Waiting time (months) (mean ± SD)	23.14 ± 22.55
Residual diuresis (litres) (mean ± SD)	0.88 ± 0.81
Dry weight (kg) (mean ± SD)	72.37 ± 14.16
Blood group (A/O/B/AB)	40.5/38.6/15.2/5.7
HLA compatibility index (mean ± SD)	12.98 ± 5.68
Last PRA	6.8 ± 17.4
Maximal PRA	12.27 ± 25.98
Onset of graft function (I/DGF/NF)	75.9/24.1/0
Induction with MP, ATG, anti-IL2-R	85.5/6.9/7.6
Maintenance immunosuppression	
CS (Y/N)	100/0
Mean daily dose ± SD (mg)	19.0 ± 3.4
CsA (Y/N)	65.8/34.2
Trough level of CsA C 0^b^ ± SD (μg/l)	278 ± 89.5
2-hour CsA post-dose level C 2^c^± SD (μg/l)	1515 ± 246
FK (Y/N)	34.2/65.8
Trough level of FK C 0^d^ ± SD (μg/l)	14.5 ± 6.4
AZA (Y/N)	38.6/61.4
Mean daily dose ± SD (mg/kg)	1.68 ± 0.36
MMF (Y/N)	61.4/38.6
Mean daily dose ± SD (mg)	1659 ± 493
Systolic BP (mean ± SD)	136.17 ± 15.41
Ca blocker/statin/ACEI or ATRA therapy	70.9/84.8/54.4
IGT-DM (Y/N)	16.9/83.1
Characteristics of the graft	
Number of arteries (1/2/3)	84.2/14.5/1.3
Number of veins (1/2/3)	90.5/8.9/0.6
Number of urethers (1/2/3)	99.4/0.6/0
Cold-ischaemia time (hours) (mean ± SD)	14.67 ± 5.43
BCS-T (mean ± SD)	0.63 ± 0.70
BSA (mean ± SD)	0.46 ± 0.52

Data are mean ± standard deviation (SD) or number (%).

W/B/A/H, White/Black/Asian/Hispanic; T/K/I/H, craniotrauma/spontaneous bleeding into the brain/intoxication/hypoxia; Y/N, yes/no; C/EC/UW/CU, Collins/Eurocollins/University Wisconsin/Custodiol; GN/TIN/DM/PCKD/AS/T, glomerulonephritis/tubulointerstitial nephritis/diabetic nephropathy/polycystic kidney disease/Alport syndrome/bilateral kidney tumour; PRA, panel reactive antibody before transplantation; I/DGF/NF, immediate/delayed/primary graft nonfunction; MP/ATG/anti-IL2R, induction with methylprednisolon/anti-thymocyte globulin/anti-IL2-R monoclonal antibody; CS, corticosteroids; AZA, azathioprine; MMF, mycophenolate mofetil; STK, systolic blood pressure; IGT-DM, impaired glucose tolerance (IGT) or diabetes mellitus in the post-transplant period; BCS-T, Banff chronicity score-transfer; BSA, Banff score of acute changes.

a CMV IgG, IgM P/N, human anti-CMV antibody detection by ELISA – positive/negative.

b CsA-C0, trough level of cyclosporin A.

c CsA-C 2, 2-hour cyclosporin A post-dose level.

d FK-C0, trough level of tacrolimus.

**Figure 2:**
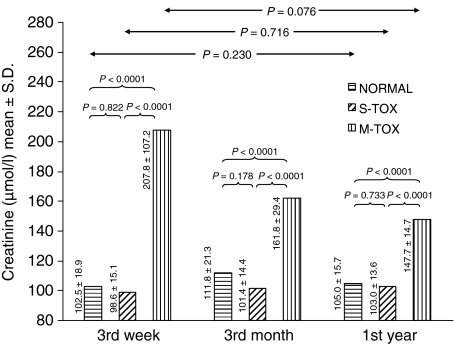
Serum creatinine of monitored groups and its evolution during one year follow-up (NORMAL, normal histological finding; S-TOX, subclinical toxicity of calcineurin inhibitors; M-TOX, manifest toxicity of calcineurin inhibitors). Data are mean ± standard deviation (SD) and level of statistical significance *P*.

Figure 2 shows the development of serum creatinine levels in individual groups. In the NORMAL and S-TOX groups, no significant change was seen in the course of the one-year monitoring. In the M-TOX group, a permanent drop in creatinine was observed, probably as a response to the repeatedly reduced CI doses; the drop reached its maximum in the period from the third week to the third month, but was not statistically significant. Changes in the glomerular filtration rate were not significant either.

### Cyclosporin A and tacrolimus toxicity

The comparisons of cyclosporin A and tacrolimus in terms of their toxic potential and ability to affect the progression of chronic changes are documented in Table 5. Comparison of data in the CsA and FK subgroups of the S-TOX and M-TOX groups was influenced by the lower number of included patients and differences in some of the parameters presented in Table 4; the results therefore cannot be considered valid. If, however, toxic signs were evaluated independently of the graft function (S+M-TOX group), no difference in respect of their influence upon the development of both toxic and chronic changes expressed by the final BCS value was identified.

**Table 5:** Comparison of Banff chronicity score of monitored groups in dependence on kind of used calcineurin inhibitor.

		3rd week		3rd month		1st year	
Group		CsA	FK^b^	CsA	FK	CsA	FK
NORMAL	*n* (%)	18 (60.0)	12 (40.0)	12 (57.1)	9 (42.9)	8 (57.1)	6 (42.9)
	CI-C0^a^ mean ± SD (μg/l)	281.3 ± 93.8	14.3 ± 3.4	202.3 ± 62.1	11.6 ± 7.5	167.6 ± 50.1	7.2 ± 1.5
	BCS mean ± SD	0.72 ± 0.75	0.58 ± 0.79	0.58 ± 0.79	0.56 ± 0.73	1.13 ± 0.35	0.83 ± 0.75
	P_BCS_	0.082		0.886		0.015	
S-TOX	*n* (%)	10 (58.8)	7 (41.2)	9 (64.3)	5 (35.7)	7 (58.3)	5 (41.7)
	CI-C0 mean ± SD (μg/l)	277.0 ± 75.0	16.4 ± 6.0	194.3 ± 55.5	8.6 ± 3.9	179.4 ± 33.4	7.8 ± 2.5
	BCS mean ± SD	0.60 ± 0.69	0.71 ± 0.76	1.22 ± 0.67	1.4 ± 0.55	1.57 ± 0.79	2.4 ± 0.55
	P_BCS_	0.343		0.738		0.115	
M-TOX	*n* (%)	8 (50.0)	8 (50.0)	7 (53.8)	6 (46.2)	6 (54.5)	5 (45.5)
	CI-C0 mean ± SD (μg/l)	296.9 ± 141.5	14.2 ± 2.4	176.3 ± 54.9	9.3 ± 1.9	153.0 ± 52.9	7.9 ± 1.3
	BCS mean ± SD	0.50 ± 0.76	0.63 ± 0.52	1.57 ± 1.13	1.83 ± 0.75	1.83 ± 0.98	2.6 ± 0.55
	P_BCS_	0.248		0.481		0.176	
S+M-TOX	*n* (%)	18 (54.5)	15 (45.5)	16 (59.3)	11 (40.7)	13 (56.5)	10 (43.5)
	CI-C0 mean ± SD (μg/l)	285.8 ± 106.4	15.2 ± 4.4	186.4 ± 54.2	8.9 ± 2.8	167.2 ± 43.7	7.9 ± 1.9
	BCS mean ± SD	0.56 ± 0.70	0.67 ± 0.62	1.38 ± 0.86	1.64 ± 0.67	1.69 ± 0.85	2.5 ± 0.53
	P_BCS_	0.295		0.515		0.123	

Data are mean ± standard deviation (SD) or number (%), and level of statistical significance P.

NORMAL, normal histological finding; S-TOX, subclinical toxicity of calcineurin inhibitors; M-TOX, manifest toxicity of calcineurin inhibitors; S+M-TOX, composite group with subclinical and manifest toxicity; BCS, Banff chronicity score; CsA, cyclosporin A.

a CI-C0, trough level of calcineurin inhibitor.

b FK, tacrolimus.

## Discussion

In this prospective study, the incidence of subclinical toxicity in repeated protocol biopsies of transplanted kidneys was monitored and we attempted to evaluate its impact upon the progression of irreversible graft changes. Of the total number of 158 biopsies conducted in the third week, signs of toxic damage were seen in 20% of biopsy samples. More than 50% of these findings were clinically silent, with normal serum creatinine levels. Only in 12% of patients, possible toxicity as the cause of graft dysfunction was signalled by the concurrent detection of elevated CI levels. Despite dose reduction, toxicity persisted in the following biopsy in the third month and in the first year in more than 80% of patients in both S-TOX and M-TOX study groups and 50% of these cases again involved clinically silent findings. CI (C 0 and C 2) levels in these groups did not differ significantly and, similar to the observations of Rush *et al.* [8], no significant difference was found in the comparison with the normal histology group, either. The detection of toxicity in grafts with normal and stabilized function thus depended upon the performance of protocol biopsy.

We employed the BCS and the evaluation of its development in all of the study groups for the assessment of pathogenic potential of these subclinical changes persisting during the first year after transplantation. A certain shortcoming of our study has been the absence of implantation biopsies which would provide more accurate information on the scope of chronic changes to the donor kidney. Yet we think that biopsies conducted in the third week provided, in this respect, adequate predictive value. As compliance with the histological sample adequacy requirement and with the exclusion criteria has been maintained, it was possible to consider the chronic changes detected in the third week donor-associated. The impact of other risk factors (Table 4), including cold ischaemia time and the delayed graft function associated therewith, was, in this short time horizon, rather theoretical. It was, however, taken into account in the same manner as in the subsequent course of the study by comparable activity in all of the studied groups. Important for the evaluation of chronic changes development, however, was the fact that the groups in the third week did not significantly differ in the level of baseline chronic changes and hence their progression and graft function alterations could be directly related to the presence of toxicity. Our findings clearly show that not only toxicity manifested by graft dysfunction but also its subclinical form resulted, compared with normal histological findings, in a significant progression of chronic changes as early as in the third month after transplantation and this trend was confirmed by further significant increases in the BCS at the end of the one-year monitoring. Interestingly, there was no significant difference in the level of chronic changes between the two toxicity groups in the third month and in the first year. A relatively pronounced progression of chronic changes in this early post-transplantation period corresponds to the findings of Chapman *et al.* [3], in whose set of protocol biopsies, the maximum of chronic changes of grafts affected by ischaemia, acute rejection or toxicity was developing in a similar pattern over the first 12 months after transplantation.

The degree of chronic changes at the end of one-year monitoring, however, was not as pronounced in this study population so as to be accompanied by significant impairment of the graft function. Nankivell *et al.* [5] also observed an increase in serum creatinine in chronic nephrotoxicity only in a period exceeding one year from the transplantation. Our evaluation of creatinine level development in relation to subclinical toxicity and progression of chronic graft changes has, moreover, been shown to be questionable, as well as the creatinine response to CI dose reduction. Despite repeated reduction, pronounced growth of the BCS accompanied by a merely insignificant increase in creatinine levels was seen in the S-TOX group. In the M-TOX group, the significant increase in the BCS was accompanied even with an apparent – albeit statistically insignificant – decrease in creatinine levels, probably because of the CI dose reduction. We therefore assume that serum creatinine does not adequately reflect the degree of graft damage especially in the early post-transplantation period [3,9], and in our study population, it was not possible to rely on changes in the glomerular filtration rate, either. This complies with the recommendation to conduct protocol biopsies not only in patients with high immunological risk to eliminate subclinical rejection [2] but also for nonrejection indications, i.e. also detection of toxicity [10], in particular during the first post-transplantation year. At present, it is not possible to avail of any adequately sensitive and specific marker or noninvasive procedure for the timely identification and further monitoring of toxicity [11,12]. The monitoring of C 2 levels, which best correlate with the area under the time-concentration curve (AUC), should reduce the risk of toxicity, yet no adequate C 2 target value has been established to date. In our study population, C 2 levels ranging within the selected therapeutic limits did not prevent the development of toxic changes, either. Moreover, a pronounced fibrogenic potential was demonstrated even with the low target AUC in month 6 and year 1 after the transplantation [13], which suggests that this approach cannot eliminate inter-individual differences in sensitivity to CI, probably genetically induced [14,15].

To date, the study population has been evaluated only for the impact of persisting toxicity upon the development of chronic histological changes over the period of one year. Nevertheless, with the significant increase in the BCS as early as in the third month after transplantation, we consider this period suitable in many cases for the adjustment of immunosuppressive therapy. The repeated CI dose reduction in our study population had only a disputable effect associated with more than 80% of persisting toxic changes in the following biopsy. Furthermore, in almost 15% of patients, it was accompanied by some form of acute rejection and further dose reduction, particularly in patients with CI levels at the bottom part of the therapeutic range, could further increase this percentage. The comparisons of cyclosporin A and tacrolimus have shown that independent of the graft function, their toxicity was similar and had comparable impact upon the progression of chronic changes in the course of the one-year monitoring. The outcomes of our study are consistent with the findings of other authors who have documented a similar impact of both CI upon the development of histological toxic changes [5] as well as upon the degree of chronic interstitial changes and level of graft function at the end of the first year after transplantation [13]. Taking this into account, a conversion to an alternative CI does not seem to be an optimal solution; a more efficient process could be – even in the case of subclinical toxicity – a conversion to any of the mTOR inhibitors, sirolimus or everolimus.

The easiest strategy resulting in the elimination of CI nephrotoxicity is, however, a complete elimination of this group from immunosuppressive protocols [16]. Positive outcomes in this respect have been presented by a comparative study documenting excellent one-year graft survival in low-risk patients with transplants from living donors, treated primarily with tacrolimus or sirolimus [17]. The safety of application of these recommendations in high-risk kidney transplant recipients, however, requires prior assessment in controlled studies. Also, until the positive effects of sirolimus or everolimus on long-term graft survival are confirmed, we believe, consistent with other authors, that CI will remain the cornerstone of immunosuppressive protocols [13] and that their administration requires optimization [18]. On the basis of study outcomes, we are, however, in favour of the option to exclude CI from therapy at the end of the third month after transplantation [19,20] by means of conversion to mTOR inhibitors in patients with repeatedly detected toxicity, low immunological risk, low baseline proteinuria [21] and negative history and current exclusion of acute rejection changes. Where these conditions are met, and regardless of the graft function and previous incidence of rejection, conversion should be probably always carried out, if toxicity persists in the first year after transplantation (Table 6). As in addition to preventing further progression of chronic damage, disappearance of the CI haemodynamic effect may be expected in this period as well as the remodelling of previously developed alterations and, in dysfunctional grafts, also function improvement [22]. In case of mTOR inhibitors contraindication is present, further CI dose reduction should be considered and conversion to tacrolimus performed, achieving concentrations close to the lower therapeutic range. Although our results suggest that tacrolimus has the same toxic potential as cyclosporin A, its more favourable metabolic profile and better anti-rejection activity may be made use of in the long-term.

**Table 6:** Calcineurin inhibitor post-transplantation toxicity – diagnostic and therapeutic algorithm.

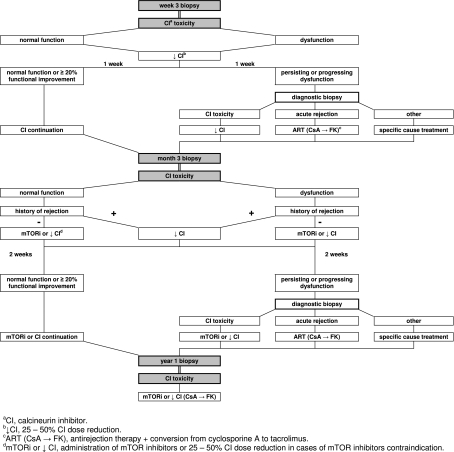

It is necessary to state that data evidencing the impact of subclinical toxicity upon the progression of chronic changes, supported by a comparison with findings from protocol biopsies in patients with normal histological results and clinically manifest toxicity, have not been published to date. Application of these findings could be of importance particularly at those workplaces where sequential biopsies of transplanted kidneys are not part of routine observational post-transplantation protocols. Sequential biopsies represent the only feasible tool allowing to detect subclinical toxicity and to control its persistence. On the other hand, it is an invasive procedure, presenting certain risks to the patient. In our set of 424 protocol biopsies in total, it was not necessary to perform any graftectomy or any surgical intervention because of haemorrhagic complications. Other serious complications have occurred only in three cases (0.7%), where two patients were given two therapeutic units of erythromass for a larger scope perirenal haematoma, one patient had to undergo superselective embolization of progressing arteriovenous parenchymal fistula with a risk of rupture. Minor complications, i.e. development of a small perirenal haematoma, transient macroscopic haematuria and minor arteriovenous fistula were reported in 27 cases (6.4%), without any further negative impact upon the patient and the fate of transplanted kidney. The incidence of clinically significant complications was hence low in our workplace and comparable to data presented in similar studies. In our opinion, the protocol which we have presented, currently supplemented with a routine conduct of implantation biopsies, seems to be adequate for the period of the first post-transplantation year in terms of the number of sequential biopsies. Our workplace avails of it with much benefit not only for the detection of toxic changes but also for the control of rejection complications arising in association with repeated reduction and minimization of CI doses or their complete elimination. It is relatively considerate to the patient and to-date preliminary results from two-year and three-year biopsies suggest that it provides sufficient space for an effective adjustment of the immunosuppressive therapy.

In conclusion, subclinical toxicity in the first year after transplantation affects a percentage of grafts comparable to that of its clinically manifest form associated with renal dysfunction. It occurs independently of dosage, blood level and type of applied CI. Even when the dose is reduced, it persists in repeated biopsies in a high percentage and results in significant progression of chronic changes as early as in the course of the first year after transplantation and hence represents an independent risk factor for chronic graft damage. Protocol biopsies seem to be a good tool for the detection of subclinical toxicity and for the control of its persistence. The impact of subclinical toxicity upon long-term graft survival, the indifferent effects of both CI and the safety of their elimination particularly in grafts with normal function, will require verification in a larger population and over a longer time span.

## References

[1] Meier-Kriesche HU, Schold JD, Kaplan B (2004). Long-term renal allograft survival: have we made significant progress or is it time to rethink our analytic and therapeutic strategies?. Am J Transplant.

[2] Rush D (2006). Protocol transplant biopsies: an underutilized tool in kidney transplantation. Clin J Am Soc Nephrol.

[3] Chapman JR, Nankivell BJ (2006). Nephrotoxicity of ciclosporin A: short-term gain, long-term pain?. Nephrol Dial Transplant.

[4] Naesens M, Lerut E, Damme BV, Vanrenterghem Y, Kuypers DR (2007). Tacrolimus exposure and evolution of renal allograft histology in the first year after transplantation. Am J Transplant.

[5] Nankivell BJ, Borrows RJ, Fung CL, O’Connell PJ, Chapman JR, Allen RD (2004). Calcineurin inhibitor nephrotoxicity: longitudinal assessment by protocol histology. Transplantation.

[6] Racusen LC, Solez K, Colvin RB (1999). The Banff 97 working classification of renal allograft pathology. Kidney Int.

[7] Solez K, Colvin RB, Racusen LC (2007). Banff ‘05 Meeting Report: differential diagnosis of chronic allograft injury and elimination of chronic allograft nephropathy (‘CAN’). Am J Transplant.

[8] Rush DN, Nickerson P, Jeffery JR, McKenna RM, Grimm PC, Gough J (1998). Protocol biopsies in renal transplantation: research tool or clinically useful?. Curr Opin Nephrol Hypertens.

[9] Viklicky O, Matl I, Voska L (2003). TGF-β1 expression and chronic allograft nephropathy in protocol kidney graft biopsy. Physiol Res.

[10] Morath C, Ritz E, Zeier M (2003). Protocol biopsy: what is the rationale and what is the evidence?. Nephrol Dial Transplant.

[11] Woywodt A, Bahlmann FH, De Groot K, Haller H, Haubitz M (2002). Circulating endothelial cells: life, death, detachment and repair of the endothelial cell layer. Nephrol Dial Transplant.

[12] Koo DD, Roberts IS, Quiroga I (2004). C4d deposition in early renal allograft protocol biopsies. Transplantation.

[13] Rowshani AT, Scholten EM, Bemelman F (2006). No difference in degree of interstitial Sirius red–stained area in serial biopsies from area under concentration-over-time curves–guided cyclosporine versus tacrolimus-treated renal transplant recipients at one zdar. J Am Soc Nephrol.

[14] Hauser IA, Schaeffeler E, Gauer S (2005). ABCB1 genotype of the donor but not of the recipient is a major risk factor for cyclosporine-related nephrotoxicity after renal transplantation. J Am Soc Nephrol.

[15] Macphee IA, Fredericks S, Mohamed M (2005). Tacrolimus pharmacogenetics: the CYP3A5*1 allele predicts low dose-normalized tacrolimus blood concentrations in whites and South Asians. Transplantation.

[16] Oberbauer R, Segoloni G, Campistol JM (2005). Early cyclosporine withdrawal from a sirolimus-based regimen results in better renal allograft survival and renal function at 48 months after transplantation. Transpl Int.

[17] Larson TS, Dean PG, Stegall MD (2006). Complete avoidance of calcineurin inhibitors in renal transplantation: a randomized trial comparing sirolimus and tacrolimus. Am J Transplant.

[18] Ekberg H, Grinyó J, Nashan B (2007). Cyclosporine sparing with mycophenolate mofetil, daclizumab and corticosteroids in renal allograft recipients: the CAESAR Study. Am J Transplant.

[19] Mota A, Arias M, Taskinen EI (2004). Sirolimus-based therapy following early cyclosporine withdrawal provides significantly improved renal histology and function at 3 years. Am J Transplant.

[20] Mulay AV, Hussain N, Fergusson D, Knoll GA (2005). Calcineurin inhibitor withdrawal from sirolimus-based therapy in kidney transplantation: a systematic review of randomized trials. Am J Transplant.

[21] Diekmann F, Budde K, Slowinski T (2008). Conversion to sirolimus for chronic allograft dysfunction: long-term results confirm predictive value of proteinuria. Transpl Int.

[22] Tostivint I, du Montcel ST, Jaudon MC (2007). Renal outcome after ciclosporin-induced nephrotoxicity. Nephrol Dial Transplant.

